# Study on the Stability of Low-Carbon Magnesium Cementitious Materials in Sulfate Erosion Environments

**DOI:** 10.3390/ma16114042

**Published:** 2023-05-29

**Authors:** Yuan Jia, Xinmei Zou, Yaoting Jiang, Yuxin Zou, Shuanglin Song, Jianyun Qin, Yongjing Wang, Lihua Zhu

**Affiliations:** 1 Hebei Provincial Laboratory of Inorganic Nonmetallic Materials, Hebei Provincial Industrial Solid Waste Comprehensive Utilization Technology Innovation Center, College of Materials Science and Engineering, North China University of Science and Technology, Tangshan 063210, China; jia132012@ncst.edu.cn (Y.J.); zouxinmei092@163.com (X.Z.); yeticu@163.com (Y.J.); zouyx0724@163.com (Y.Z.); 2 State Key Laboratory of Coal Mine Safety Technology, China Coal Technology & Engineering Group, Shenyang Research Institute, Fushun 113122, China; wangyongjing_85@163.com; 3 Kuqa County Yushuling Coal Mine Co., Ltd., Kuqa 842099, China; qinjianyun2023@163.com; 4 School of Civil Engineering, Hebei University of Engineering, Handan 056038, China; zhulihuahd@163.com

**Keywords:** hydrated magnesium silicate, sulfate erosion, dry-wet circulation, cement

## Abstract

The current investigation focuses on the stability of the magnesium oxide-based cementitious system under the action of sulfate attack and the dry-wet cycle. The phase change in the magnesium oxide-based cementitious system was quantitatively analyzed by X-ray diffraction, combined with thermogravimetry/derivative thermogravimetry and scanning electron microscope, to explore its erosion behavior under an erosion environment. The results revealed that, in the fully reactive magnesium oxide-based cementitious system under the environment of high concentration sulfate erosion, there was only magnesium silicate hydrate gel formation and no other phase; however, the reaction process of the incomplete magnesium oxide-based cementitious system was delayed, but not inhibited, by the environment of high-concentration sulfate, and it tended to turn completely into a magnesium silicate hydrate gel. The magnesium silicate hydrate sample outperformed the cement sample, in terms of stability in a high-concentration sulfate erosion environment, but it tended to degrade considerably more rapidly, and to a greater extent, than Portland cement, in both dry and wet sulfate cycle environments.

## 1. Introduction

In response to China’s environmental policy, the development of new cementitious materials with complementary properties and environmental characteristics of traditional cement has become the research direction of the low-carbon cement industry, and, in this direction, magnesium oxide-based cementitious materials are of great importance. As a new type of green high-performance building material [1], magnesium oxide-based cementitious materials not only have good cementitious properties, but also have the characteristics of a low-pH alkali environment, high strength, and fast molding; besides, it is widely used in the curing of heavy metal ions and the comprehensive utilization of industrial solid wastes [2,3,4,5]. The development and research of magnesium oxide-based cementitious materials can aid in the rational utilization of abundant magnesium-based resources and idle industrial wastes, which are in line with China’s national strategy for sustainable development, and have a wide range of potential applications.

Sulfate erosion on concrete is a kind of corrosive medium damage with significant deterioration, and it is also one of the important factors affecting the durability of concrete; at the same time, it is a kind of environmental water erosion, with the most complex and harmful factors [6,7,8]. Scholars from all over the world have been devoted to studying and exploring the erosion process and erosion mechanism of cement concrete in different erosion media [9,10,11,12,13], constantly exploring new efficient and environmentally friendly cementitious materials to seek ways to improve the erosion resistance of concrete. In this context, there is an urgent need for a new type of high-performance building material to cope with sulfate attacks, and to improve the cement matrix’s sulfate resistance.

Large amounts of sulfate in the soils of the coastal and western salt lake regions, as well as in seawater, groundwater, and spring waters [14,15,16,17,18,19], will cause sulfate erosion of the cementitious material and, hence, aggregate exposure, sand, expansion, and collapse, which will seriously affect the performance of cementitious structures [20]. Sulfate erosion of concrete structures in roads, bridges, hydroelectric engineering, and building foundations has become a major issue in recent years [21]. Cement-based buildings, especially in coastal areas, and in prolonged contact with sulfate-rich seawater, are extremely susceptible to corrosion by sulfuric acid and other acid salts. Related sulfate erosion causes deterioration of the structure and cement matrix, to the extent that concrete structures are damaged, causing major hidden safety risks and increasing maintenance costs [22,23]. Direct interaction between sulfuric acid ions and the structure causes a concrete damage peeling phenomenon, which contradicts our nation’s sustainable development objective.

The development of the cement industry is not aligned with the country’s carbon peaking and carbon neutrality goals. Research on alternative gel materials to replace cement has become a priority, due to the urgency in improving concrete’s resistance against sulfate erosion, which leads to corrosion damage. This phenomenon is most common in coastal and inland salt lake areas, where acidic groundwater and high-viscosity soil environments contain sulfates. The study of a novel gel material in this paper should be applied to real-life situations, and, hence, research on its performance in sulfate erosion environments is essential. The corrosion behavior of magnesium-based cementite samples in the environment of high concentration sulfate and sulfate dry-wet cycle erosion was studied and compared with that of ordinary Portland cement.

## 2. Materials and Methods

### 2.1. Materials

Magnesia Specialties (Martin Marietta, LLC, Raleigh, NC, USA), manufactured using lightly fired, mechanically milled magnesium oxide (MgO) and silica fume (SF) was prepared by Elkem AS Silicon Materials in China. As cement and aggregate, ordinary Portland cement (PO 42.5) and quartz sand, produced by the Minghai Environmental Protection Quartz Sand Factory, which met the Chinese national standard GB175-2007, were used. The chemicals used were sodium hexametaphosphate ((NaPO_3_)_6_) and anhydrous sodium sulfate (Na_2_SO_4_), both produced by Sinoptric Chemical Reagent Co., LTD. in Shanghai, China.

The chemical composition and physical properties of magnesium oxide and silica fume are represented in Table 1:

### 2.2. Sample Preparation and Test Process

Magnesium silicate hydrate specimen (MS): quartz sand was used as aggregate; mix ratios are shown in Table 2. The raw materials were weighed accurately, according to the mix ratio. Production of specimens included the following stages: At first, (NaPO_3_)_6_ (Na-HMP) was dissolved in water and stirred to accelerate the dissolution, then poured into the stirring pot [24]. Then, the active MgO powder and silica fume were mixed (nMgO:nSiO_2_ = 1:1) and introduced evenly to the mixture in several steps. The cement mortar mixer was used to stir the mixture to a uniform state. After completing the mixing stage, the evenly mixed mortar was placed in the mold, and compaction was performed with the vibration table to eliminate the bubbles in the test block. At the same time, the surface was scraped flat, a good mark was made, and the samples were left in the laboratory for 24 h, before demolding. The mortar specimen, after demolding, was put into the standard cement curing chamber for 56 d, until the execution of experiments.

Ordinary Portland cement specimen: the raw materials were accurately weighed, according to the mix ratio in Table 2. First of all, water was poured into the mixing pot of the cement mortar mixer; then, the OPC was added. Following, the quartz sand was poured into the sand-loading device above the mixer, and the automatic gear was opened. The mixing time was equal to that of hydrated magnesium silicate, until the mortar was stirred evenly. The molding process was the same as the MS specimen production, in which it was inserted into the vibration table many times to eliminate bubbles, so as not to affect the strength of the specimen in the later stage; after the specimen was released, it was placed in the standard cement curing room for 56 d, until the test.

Northwest China is one of the areas with serious salinization, where there are large salt lakes. These salt lakes contain a lot of corrosive ions, such as chloride ions, sulfate ions, etc. [25,26,27,28]; thus, a high sulfate concentration solution was used in the current study. The uncured (NC-MS) MS specimens were divided into two groups (NC-MS-1 and NC-MS-2). Distilled water was used in the NC-MS-1 group, and 20% Na_2_SO_4_ solution was used in the NC-MS-2 group (sodium sulfate solution was replaced once a week), to ensure that the liquid level could not cross the top of the specimen. In order to prevent water evaporation and ensure that the concentration of the ionic solution in the solution remained unchanged [29], the container was capped, and the soaking solution was changed every 7 days. In the middle of the process, the NC-MS-1 sample, at the curing age of 56 d (calculated from soaking in sodium sulfate solution), was moved into a sodium sulfate solution with 20% mass concentration (sodium sulfate solution was changed once a week) for further soaking, as shown in Table 3.

To maintain the testing of 56 d (C-MS) of the specimen (MS and OPC mortar specimen), each was divided into two groups as follows: (i) in the mass concentration of distilled water and 20% sodium sulfate solution (once every 7 days, replacement soaking solution; at the same time, the experimental group of bubble water in water), (ii) and sulfate in the dry-wet circulation tester (in a 5% mass concentration of sodium sulfate solution, with the settings parameter as follows: soaking time was 15 h, air drying time was 1 h, drying time was 6 h, cooling time was 2 h, drying temperature was 80 °C, cooling temperature was 25 °C, and the time to complete a dry and wet cycle was 24 h).

The specimens were taken from the soaking solution and the testing machine’s wetting and drying cycle, with the same number of blocks as the prescribed age, and the surface moisture, was wiped dry. The surface state was observed and recorded, the compressive strength of the fractured part of the specimen was tested, and the data was recorded. The compression rate of the press was controlled in the range of 1.6–2.4 kN/s. Each data group was recorded, and individual data with particularly large deviations were removed. The final result was averaged after conversion.

### 2.3. Test Method

The samples were characterized by X-ray diffractometer (XRD, D8 advance X-ray diffractometer, Brooke AXS, Germany, XRD D/Max 2400 V diffractometer, Cu Kα radiation, scanning rate 10°/min), thermogravimetric analyzer (TGA/DTG, alumina crucible, sample heating at a rate of 10 °C/min, in a nitrogen atmosphere of 50~1000 °C), and field emission scanning electron microscope (SEM-EDAX test before drying and gold plating).

The compressive strength refers to the maximum pressure that the material can withstand without lateral restraint. The specific test method is to place the test block in the center of the pressure plate and press it perpendicular to the pressure surface at a speed of 0.5 kN/s until the test block is destroyed. The load at this time is recorded, and the compressive strength of the test block is calculated, according to the following formula:

$$f=\frac{F}{S}$$

where f is compressive strength, MPa; F is the failure load of the specimen, N; S is the stress area of the specimen, mm^2^.

## 3. Results and Discussion

### 3.1. Effect of Sulfate Erosion Environment on Hydration Mechanism of Magnesium-Based Cementitious Materials

**Figure 1:**
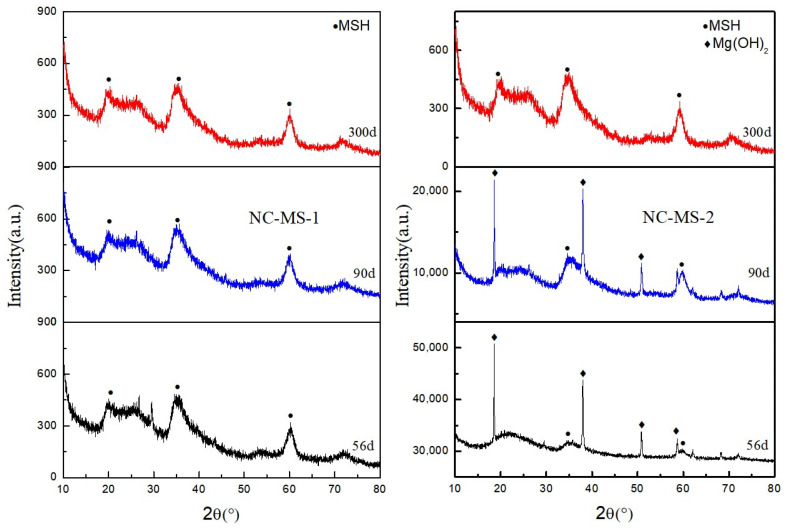
Phase changes of uncured MS specimens in different solutions at different ages.

In the process of specimen production, a part of NC-MS net slurry specimens was soaked in distilled water and Na_2_SO_4_ solution for 56 d, 90 d, and 300 d, respectively. XRD results of the related specimens are demonstrated in Figure 1. According to the findings, the formation of a new crystal peak was not detected in the XRD pattern. After analysis, it was found that, at the curing age 56 d, NC-MS-1 completely reacted to generate magnesium silicate hydrate (MSH), and there was a large amount of Mg(OH)_2_ in NC-MS-2. After 90 days, the phase of the NC-MS-1 sample did not change, and there was still some Mg(OH)_2_ in the NC-MS-2 sample. After curing for 300 d, NC-MS-1 still new phase formation was not observed, and all NC-MS-2 phases reacted into MSH.

MSH was finally generated in the solution immersion, and no new products were generated in the erosion environment. It can be demonstrated that the hydration products of magnesium oxide-based cementitious materials did not react with SO_4_^2−^, the formation and reaction trends were not apparent, and the development of MSH was relatively slow, since the sulfate solution only delayed the internal reaction process. Notwithstanding that, these findings revealed the necessity of further investigation with other test methods to determine whether new substances were produced or not.

**Figure 2:**
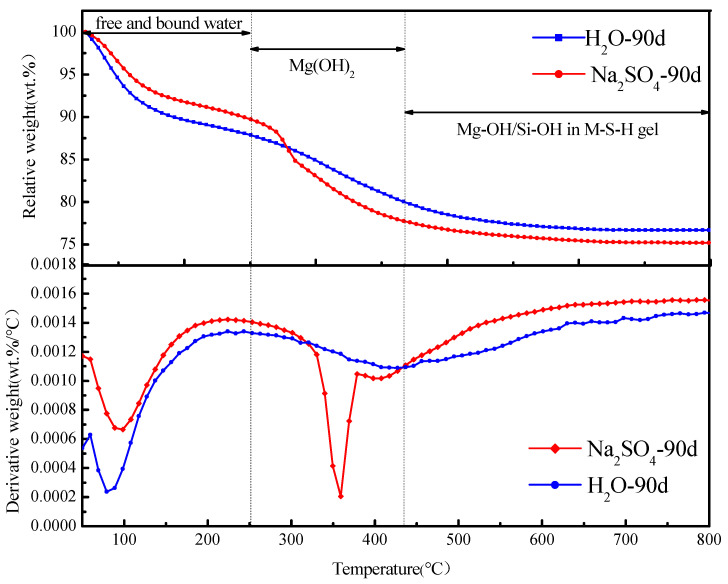
TGA and DTG curves of MS specimens soaked in Na_2_SO_4_ solution for 90 days.

Thermal gravimetric analysis was combined with TGA and DTG curves, and the results are shown in Figure 2. The sample weight-loss curve can be divided into three main stages [30]: The first stage is 50~250 °C, and the free and bound water in the reaction products are evaporated in this stage. In the second stage, 250~430 °C, the cause of weight-loss is the decomposition of Mg(OH)_2_. The third stage is 430~800 °C, in which the structural water in MSH is removed.

In Figure 2, the thermal weight-loss trend of magnesium oxide-based cementitious materials under the two treatment environments was observed, and the curves were consistent, indicating that the weight-loss of MSH under the two environments was the same; hence, there was no significant difference in the reaction products generated. The MS specimen, under a sulfate erosion environment, had a significant weight-loss at about 430 °C, which may be influenced by sulfate in the process of converting Mg(OH)_2_ to MSH by the reaction between Mg(OH)_2_ and silica fume, resulting in a large residual amount of Mg(OH)_2_ at this stage, resulting in water-loss, which was consistent with the results of XRD analysis. Mg(OH)_2_ was primarily converted to MSH in a water environment, while the reaction process was prolonged in a sulfate solution; therefore, a small residual remained.

The chemical reaction of the magnesium-based cementitious system was analyzed by thermodynamic calculation. Under standard conditions, (101.3 kPa and 298 K) represents the standard molar entropy function (the specified entropy of 1 mol of a pure substance), the standard molar generation function (the change in enthalpy of reaction from the most stable elemental to 1 mol of the pure compound), and the standard molar Gibbs free energy (the free energy from stable elemental to 1 mol of a compound or unstable elemental and other forms of substance), respectively (Table 4) [31,32]. The possible chemical reactions of the magnesium-based cementitious system immersed in Na_2_SO_4_ solution are shown in Table 5.

Equations (1)–(3) were used to calculate the thermodynamics of chemical reactions in the reaction system.

(1) SθΔf=∑iViSmθ

(2) HθΔf=∑iViHmθΔf

(3) GθΔf=∑iViGmθΔf=−RTlnK

where, the value of K is a constant, which indicates the reaction degree and stoichiometric reaction coefficient of chemical reaction. R = 8.314 J/(mol · K); T depicts the thermodynamic temperature in Kelvin (K). SθΔf>0 indicates that the reactions are going to equilibrium; HθΔf<0 indicates that the chemical reaction is exothermic and can be carried out spontaneously under natural conditions. GθΔf<0 indicates that a chemical reaction can proceed spontaneously under natural conditions, and, the smaller the value, the easier the reaction.

According to the calculation of thermodynamic parameter data in Table 4, it can be seen that 3MgO·2SiO_2_·2H_2_O (Chrysotile) and 3MgO·4SiO_2_·H_2_O (Talc) may be generated when MgO and SiO_2_ are mixed with H_2_O. According to the calculation and comparison, reacting with Na_2_SO_4_ in a magnesium oxide-based cementitious system was difficult. The Gibbs free energies of MgSO_4_·6H_2_O, MgSO_4_·7H_2_O, and Na_2_SiO_3_ that the reaction may generate are significantly higher than those of Mg^2+^, SiO_2_, and Na_2_SO_4_ as reactants; it was further demonstrated that SO_4_^2−^ did not participate in the reaction process of the magnesium-based cementitious system, and the experimental results were consistent with the XRD phase analysis.

Based on the above analysis, it can be concluded that the magnesium silicate hydrate gel, after curing for 56 d, is relatively stable, and the magnesium silicate hydrate specimens, after curing for 56 d, are used in the following tests.

### 3.2. Effects of Long-Term Sulfate Erosion Environment on Different Cementification Systems

#### 3.2.1. Comparison of Macroscopic Morphology

**Figure 3:**
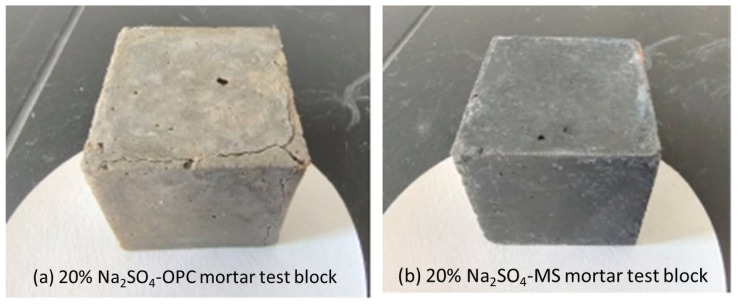
Morphology of specimens soaked in Na_2_SO_4_ solution for 270 d.

The sulfate immersion test is a very slow erosion process, and the specimen’s appearance has no obvious erosion change in a short time [34]. When the two groups of samples were immersed in the solution for 270 d, the apparent morphology of the MS mortar samples was not damaged. This reflects, to a certain extent, that the magnesium oxide-based cementitious material is relatively stable in the sulfate environment, and the erosion environment does not cause serious damage to the MS mortar test block, whereas cracks appeared in the OPC mortar samples when they reached a certain erosion age in the Na_2_SO_4_ solution, as shown in Figure 3. The integrity was severely damaged, due to the increase in erosion time, which also caused the cracks to widen gradually and develop into a series of clearly visible cracks and some corrosion pits of various sizes. These changes not only negatively impacted the appearance, but also the durability and usability.

#### 3.2.2. Comparison of Microscopic Morphology

**Figure 4:**
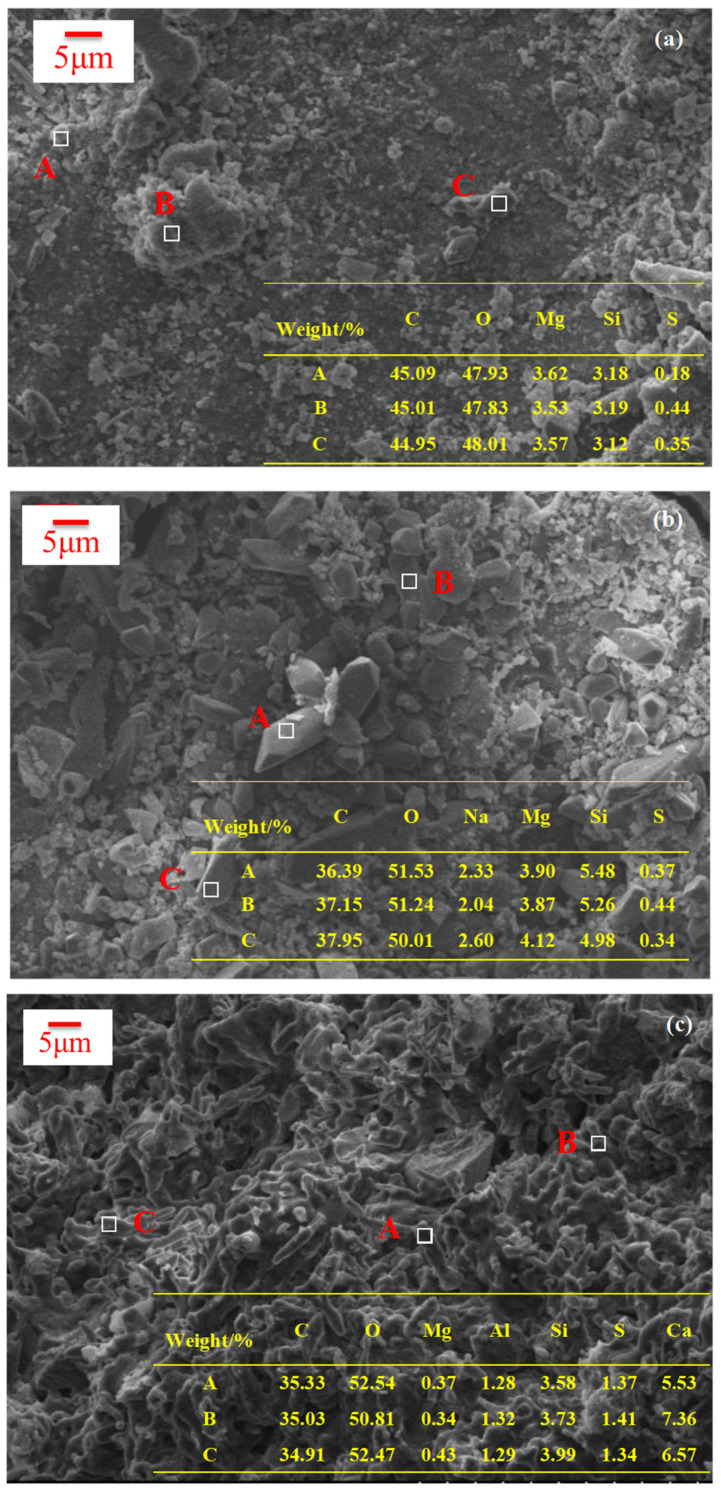

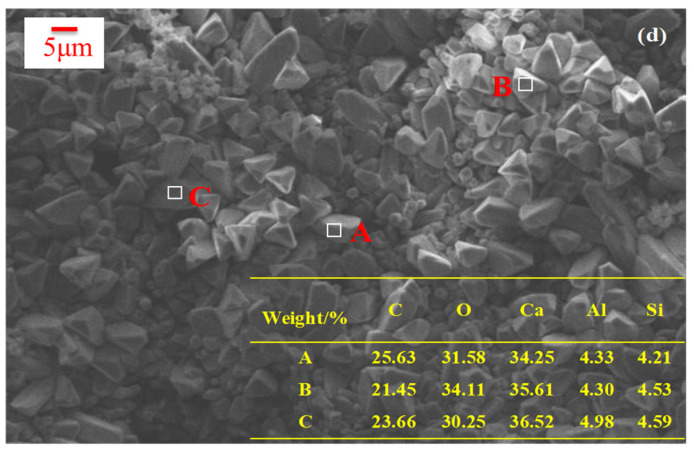
SEM image of the specimen immersed in sulfate erosion solution for a long time. (**a**) MS mortar specimen before erosion; (**b**) MS mortar specimen after erosion; (**c**) OPC mortar specimen before erosion; (**d**) OPC mortar specimen after erosion.

Before and after erosion, SEM images of the magnesium silicate hydrate sample and ordinary Portland cement sample completely immersed in 20% Na_2_SO_4_ solution are shown in Figure 4 (the table shows the element ratio diagram of the three-point fixed-point analysis energy spectrum).

The samples of magnesium silicate hydrate cement mortar soaked in a sodium sulfate solution were subjected to X-ray energy dispersive spectroscopy (EDS) examination, in conjunction with SEM pictures. It can be seen from Figure 4a,b that, before and after erosion, the immersed magnesium silicate hydrate cement mortar did not change much. Sodium crystallizes on the surface of the specimen, which might be due to the accumulation of sodium on the surface when the specimen is soaked for a long time, but there is no change in the interior. From the weight analysis aspect, it can be seen that the content of the sulfur element was tiny, indicating that SO_4_^2−^ did not react with the hydration product of magnesium silicate cement hydrate and did not exist in the form of an erosion product.

Compared with magnesium silicate hydrate specimens under the same conditions, the following are the phase changes of OPC specimens before and after erosion. Combined with EDS analysis, CaCO_3_ is generated on the surface of the cement. For 20% Na_2_SO_4_ solution, there are many pores in the whole cement area, and the initial microcrack expands. In general, erosion products are thought to result in destructive expansion [35]. Numerous studies have demonstrated that calcium vanadate is created when cement hydration products and sulfate react [36,37,38]. Filling in micropores and microcracks with erosion products enhances concrete’s compactness [39]. If the erosion products are formed in a large enough space, the crystal growth will not approach the threshold for generating expansion stress, and the concrete performance will not degrade. However, cracks will appear when the attack products’ expansion stress is greater than the tensile stress of the concrete.

Another factor is in the process of sulfate erosion, often inevitably affected by the carbonization of carbon dioxide in the air. Cement degradation is accelerated by the combined effects of carbonation and sulfate, as the carbonization reaction of ettringite, or generation of SO_4_^2−^ sulfur aluminate decomposition. Therefore, dissolved in pore solution, and through the concentration diffusion migration to the carbonization zone, carbonization zone erosion does not occur in advance, and carbonization of tricalcium aluminate reaction and generate ettringite and sulfur aluminate, because such kinds of decomposition and formation of cycles push for cement carbide internal development [40]. When the expansion stress is large, microcracks will occur inside the cement. Carbonation reaction can accelerate the diffusion of sulfate in cement, thus reducing the sulfate resistance.

#### 3.2.3. Mechanical Properties Analysis

The compressive strength of the two specimens is demonstrated in Table 6:

In this experiment, the sulfate resistance coefficient was calculated using the ratio of the compressive strengths of the specimens soaked in H_2_O and Na_2_SO_4_ [26], and the degree of change in the corrosion resistance coefficient with erosion time was used to characterize the sulfate resistance of the specimens. The compressive corrosion resistance coefficient of 56 d (treatment time is 0 d) was 1.

**Figure 5:**
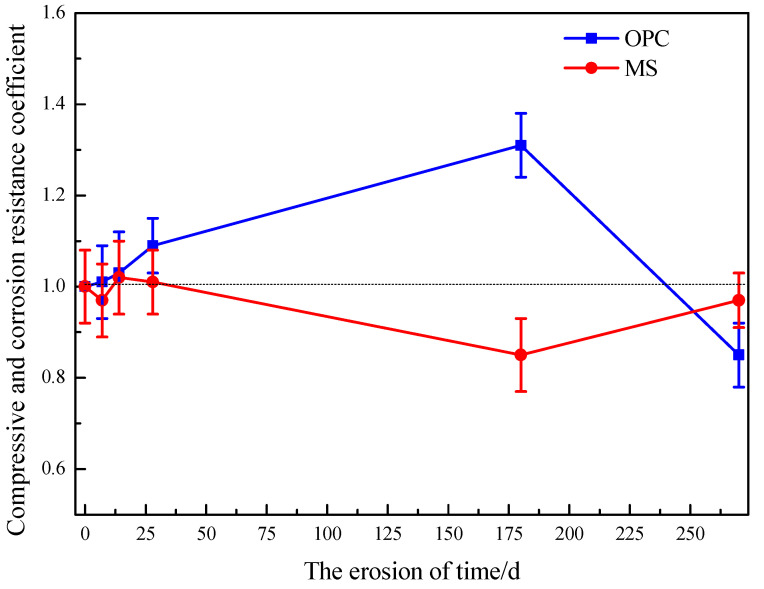
Resistance coefficient of two kinds of cement to sulfate.

The sample’s compressive strength and corrosion resistance coefficient were calculated according to the equation below:

(4) $$K_{f}=100\%\times \frac{f_{liquid}}{f_{water}}$$

where the compressive strength corrosion resistance coefficient (%); *f_liquid_* is the compressive strength (MPa) of the specimen under the dry-wet cycle in erosion solution; *f_water_* is the compressive strength (MPa) of the specimen immersed in water at the same age. The results obtained are shown in Figure 5.

Combined with the compressive strength values in Table 6, the compressive strength of the MS mortar specimen in sulfate solution did not increase or decrease significantly compared with that in H_2_O at the same age, and its compressive and corrosion resistance coefficient fluctuated around 1 and changed stably, which indicated that the MS mortar specimen has good resistance to sulfate erosion.

When OPC mortar specimens were exposed to sulfate, the compressive strength and compressive corrosion resistance coefficient first increased and subsequently fell, with the compressive corrosion resistance coefficient dramatically declining in the final stages of erosion. In regards to strength, along with the change of soaking age in Table 6, it can be seen that sulfate had a significant impact on the OPC specimen; in sulfate solution, the previous, within 28 d compressive corrosion resistant coefficient, increased gradually, and specimen compressive strength was greater in the sulfate than in the water. This is because the SO_4_^2−^ in sulfate can react with the hydration products of cement. Expandable materials, such as gypsum and ettringite, are formed, which fills the internal pores and reduces the overall porosity [41]. However, microdamage and cracks in the concrete will develop when there is too much swelling material and not enough space, causing microcracks and loss of strength [42]. When the erosion age reached 270 d, several cracks extended from the edges and corners of the specimen surface, and the integrity was destroyed. The compressive strength gradually decreased at this time, and the compressive and corrosion resistance coefficient was far less than 1. However, because the specimens were involved in the hydration reaction throughout this phase, the strength of the specimens in water increased with the expansion of immersion age. The water environment is conducive to the reaction, and the hydration products generated make the specimens have greater strength [13]. The compressive strength of MS mortar specimens immersed in Na_2_SO_4_ was relatively stable, and the compressive strength did not change significantly with erosion, which may be because the hydration products of magnesium oxide-based cementing materials would not react with SO_4_^2−^.

### 3.3. Effect of Sulfate Dry-Wet Cycle Erosion Environment on Different Cementitious Systems

#### 3.3.1. Surface Damage of the Specimen

**Figure 6:**
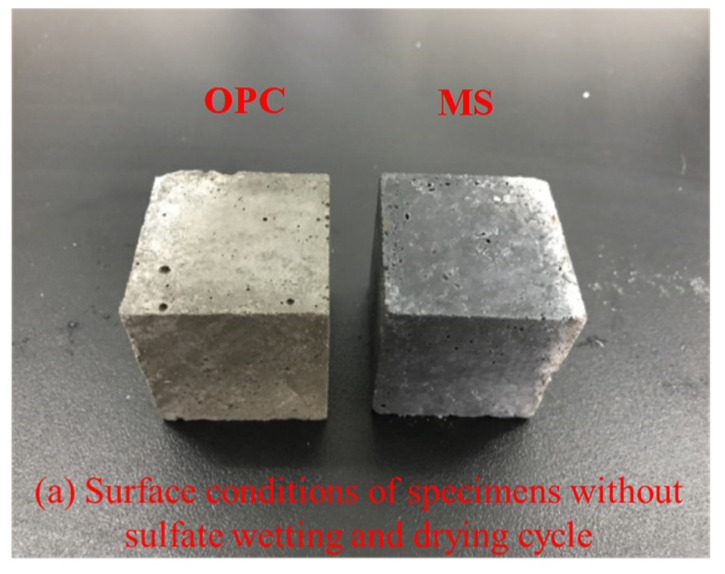

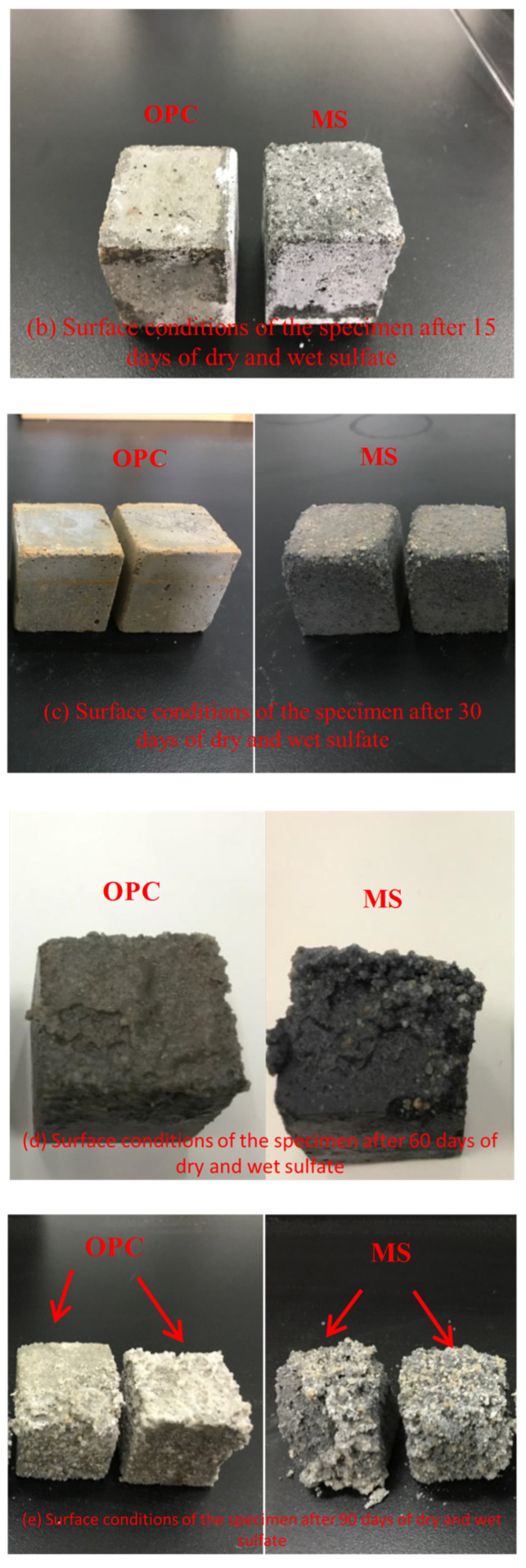
The apparent morphology of the test block after the drying and wetting cycle with sulfate.

The surface of the specimen showed specific characteristics after different sulfate drying and wetting cycles: In sulfate dry-wet cycle 15 d, the MS specimen surface had a salting-out phenomenon; this was because of the drying conditions in the dry-wet cycle test machine, the heat on the surface of the block causing sulfate solution evaporation, and increasing solution concentration; when it reached its saturation, sulfate crystals could precipitate, thus leaving a layer of white on the block surface layer of salt, as seen in Figure 6b. When the dry and wet sulfate cycle reached 30 days, it could be observed that the MS specimen had begun to be destroyed; the corner position fell off, exposing the aggregate, and the surface glue was dissolved by sulfate in the process of the dry and wet sulfate cycle, as seen in Figure 6c. At the end of 60 days, the MS test block had been sanded, the integrity was destroyed, and the degree of erosion was deepened. The expansion stress generated by physical and chemical reactions inside the OPC mortar specimen was greater than the critical value of tensile stress of the specimen, resulting in expansion cracks, which sped up the invasion rate of sulfate into the specimen, and so on, as seen in Figure 6d. At 90 d, the specimens were completely destroyed, the glue was dissolved, the aggregate was peeled off, and the mass loss was serious. The damage degree of MS mortar specimens was greater. From the apparent morphology in Figure 6e, it could be seen that MS specimens were relatively influenced by the wetting and drying cycle.

#### 3.3.2. Mechanical Properties Analysis

**Figure 7:**
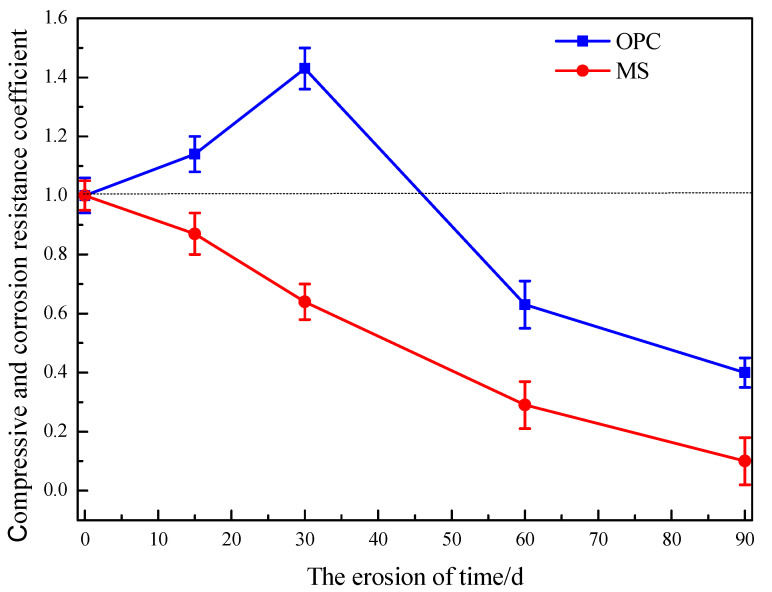
Resistance coefficient of two kinds of cement to wet and dry cycle of sulfate.

The compressive strength of the two cement mortar test blocks in the sulfate dry and wet circulation environment is shown in Table 7. A line graph was created with the data in Table 7, as shown in Figure 7.

It can be seen from Table 7 that the strength of the MS mortar specimen was higher than that of the OPC mortar test block in the early stage of sulfate dry-wet cycle erosion. With the continuous erosion, the compressive strength of the MS specimen reached 43.02 MPa at 30 d, which was much higher than the compressive strength value of OPC in the same period. The strength of OPC specimens increased gradually with the hydration reaction and reached 51.86 MPa on the 90th day, which was higher than that of MS specimens. However, even soaking in water, the strength of MS specimens remained steady and did not change, thanks to their unique structure and significant internal reaction process.

OPC’s strength increased first, and then decreased after the sulfate dry-wet cycle erosion treatment [43,44], which was due to the reaction between the specimen and SO_4_^2−^. SO_4_^2−^ was enriched in the interphase transition zone, generating expansion products, such as gypsum and ettringite. In the early stage, due to the infiltration of less sulfate solution, the generated products filled the internal voids. In the later stage, with the increase of erosion degree, the expansion material continued to form, and the reaction products inside the specimen kept increasing; thus, the volume kept increasing. At this stage, the strength gradually increased. When the crystallization pressure, caused by ettringite and gypsum, is transmitted to the cement matrix [45], the original microcracks will gradually expand, accelerating the intrusion of sulfate solution. This process continues to cycle, coupled with the alternating dry and wet cycle of the environment in which the specimen is located. Then, the final microcrack propagates into cracks, which is the overall failure of the specimen, so the later strength decreased sharply.

When the MS mortar specimen was carried out for 30 d, its strength decreased significantly, compared with that of 15 d. At 60 d, the specimen was completely destroyed, and its strength decreased significantly, indicating that the environment had a great influence on the MS specimen. In the drying stage, the high temperature destroyed the bound water in the magnesium-based cementing system, and the internal structure of the specimen was damaged due to the drying water loss, which decreased the strength of the specimen. At the same time, the hydration products of OPC are relatively little affected. With the sulfate attack and the wetting and drying cycle, the pores and cracks of the interface were expanded gradually. The transfer of SO_4_^2−^ to the cement surface was carried out under the condition of water saturation. In an environment with frequent dry-wet cycles, the corrosive solution penetrates more easily. Finally, it expanded, cracked, and fell off, reducing the strength and stability of the test block.

## 4. Conclusions

The purpose of this study is to study the effects of the dry-wet cycle and sulfate attack on the stability of new cementitious materials, to study the macroscopic and microscopic phenomena of OPC and magnesium oxide-based cementitious materials in a sulfate environment, and to compare the compressive strength. The conclusions are summarized as follows:

1. MS specimens cured for 56 d were more stable than uncured specimens soaked in a sulfate solution. The reaction process of magnesium oxide-based cementified materials could be delayed, to a certain extent, under the environment of sulfate erosion, but, after curing for 300 days, nevertheless, it completely reacted into magnesium silicate hydrate gel.
2. SO_4_^2−^ did not react with hydration products of magnesium silicate hydrate gel, so MS specimens were more stable than OPC specimens under a sulfate erosion environment. However, its compressive strength was not as good as the OPC sample.
3. Under the condition of dry and wet cycle sulfate erosion, the damage degree of magnesium silicate hydrate was obvious, and the strength loss was large, which might be due to the destruction of the structure of the magnesium oxide-based cementing system under the condition of dry water loss, which evaporates the internal bound water and causes the damage of the specimen.

The results show that, under the goal of a green, low-carbon, and circular economy, the development of new cementitious materials has become a trend in the field of building materials, and improving its compressive strength has become an urgent problem to be solved.

## Figures and Tables

**Table 1:** Basic chemical compositions of MgO and SF (wt.%).

	MgO	CaO	SiO_2_	Fe_2_O_3_	Al_2_O_3_	Na_2_O	K_2_O	SO_3_	Cl	LOI
MgO	98.21	0.89	0.36	0.05	0.19	-	-	0.02	0.55	1.7
SF	1.12	0.59	95.9	0.40	0.24	0.38	1.64	0.27	-	2.0

**Table 2:** Mix ratio of different mortar blocks in sulfate immersion.

Cement Type	Water-Cement Ratio	Cement-Sand Ratio	Admixture
MS	1:2	1:1	Na-HMP
OPC	1:2	1:1	-

**Table 3:** Two kinds of specimens soaked in different solutions at different periods.

Soaking Duration (d)	0–56	56–90	90–300
NC-MS-1	H_2_O	Na_2_SO_4_	Na_2_SO_4_
NC-MS-2	Na_2_SO_4_	Na_2_SO_4_	Na_2_SO_4_

**Table 4:** Thermodynamic parameters of phase in magnesium oxide-based cementitious system [33].

Minerals or Species	$S_{m}^{\theta }$ (kJ/mol)	HmθΔf (kJ/mol)	GmθΔf (kJ/mol)
MgO(S)	26.95	−601.50	−569.23
Mg(OH)_2_(S)	63.14	−924.54	−833.56
M_3_S_2_H_2_(Chrysotile)	221.30	−4361.66	−4034.24
M_3_S_4_H(Talc)	260.80	−5915.90	−5536.27
Mg^2+^(aq)	−138.00	−466.85	−454.89
OH^−^(aq)	−10.71	−230.03	−157.34
H^+^(aq)	0.00	0.00	0.00
SiO_2_(glassy state)	47.41	−903.20	−850.59
H_2_O(l)	69.95	−285.83	−237.19
H_3_SiO_4_^−^(aq)	112.55	−1426.16	−1253.98
H_2_SiO_4_^2−^(aq)	−12.97	−1396.62	−1187.02
Na_2_SO_4_(aq)	149.49	−1387.11	−1269.35
SO_4_^2−^(aq)	20.00	−909.27	−744.48
Na^+^(aq)	58.41	−240.30	−261.88
MgSO_4_·6H_2_O(crystalline state)	348.11	−3085.99	−2631.24
MgSO_4_·7H_2_O(crystalline state)	372.00	−3388.70	−2871.58
Na_2_SiO_3_(s)	113.81	−1557.62	−1463.65

**Table 5:** Thermodynamic parameters of possible chemical reactions in magnesium oxide-based cementitious systems.

ID	Chemical Reaction	$S_{m}^{\theta }$ (kJ/mol)	HmθΔf (kJ/mol)	GmθΔf (kJ/mol)
1	MgO + 2H^+^ → Mg^2+^ + H_2_O	−95.00	−151.18	−122.50
2	SiO_2_ + 2OH^−^ → H_2_SiO_4_^2−^	−38.95	−33.38	−21.75
3	SiO_2_ + OH^−^ + H_2_O → H_3_SiO_4_^−^	5.91	−7.11	−8.86
4	Mg^2+^ + 2OH^−^ → Mg(OH)_2_	222.56	2.35	−63.99
5	3Mg(OH)_2_ + 2SiO_2_ → 3MgO·2SiO_2_·2H_2_O + H_2_O	7.03	−67.47	−69.57
6	3Mg(OH)_2_ + 4SiO_2_ → 3MgO·4SiO_2_·H_2_O + 2H_2_O	21.67	−101.14	−107.60
7	3MgO + 2H_2_O + 2SiO_2_ → 3MgO·2SiO_2_·2H_2_O	−94.26	−179.10	−151.00
8	3MgO + H_2_O + 4SiO_2_ → 3MgO·4SiO_2_·H_2_O	−79.62	−212.77	−189.03
9	MgO + SiO_2_ + Na_2_SO_4_ + 6H_2_O → MgSO_4_·6H_2_O + Na_2_SiO_3_	−181.63	−36.82	17.42
	MgO + SiO_2_ + Na_2_SO_4_ + 7H_2_O → MgSO_4_·7H_2_O + Na_2_SiO_3_	−227.69	−53.7	14.27

**Table 6:** Compressive strength of specimens.

Time (d)	OPC Mortar Test Block (MPa)			MS Mortar Test Block (MPa)		
Maintenance of 56 d	58.76			57.44		
Process Started	Water Immersion	Na_2_SO_4_ Soak	Compression and Corrosion Resistance Coefficient (Kf)	Water Immersion	Na_2_SO_4_ Soak	Compression and Corrosion Resistance Coefficient (Kf)
7 d	60.32	60.86	1.01 (±0.08)	61.82	60.32	0.97 (±0.08)
14 d	61.58	63.45	1.03 (±0.09)	62.43	63.48	1.02 (±0.08)
28 d	64.28	69.76	1.09 (±0.06)	62.63	63.54	1.01 (±0.07)
180 d	50.25	65.73	1.31 (±0.07)	60.19	54.91	0.85 (±0.08)
270 d	68.62	58.37	0.85 (±0.07)	61.86	60.13	0.97 (±0.06)

**Table 7:** Compressive strength of specimens under different conditions.

Time (d)	OPC Mortar Test Block (MPa)			MS Mortar Test Block (MPa)		
Maintenance of 56 d	58.76			57.44		
Process Started	Water Immersion	Na_2_SO_4_ Soak	Compression and Corrosion Resistance Coefficient (Kf)	Water Immersion	Na_2_SO_4_ Soak	Compression and Corrosion Resistance Coefficient (Kf)
15 d	34.10	38.73	1.14 (±0.06)	46.25	40.10	0.87 (±0.07)
30 d	39.98	57.02	1.43 (±0.07)	43.02	27.53	0.64 (±0.06)
60 d	50.80	32.24	0.63 (±0.08)	44.48	12.89	0.29 (±0.08)
90 d	51.86	20.81	0.40 (±0.05)	45.76	4.66	0.10 (±0.08)

## Data Availability

Not applicable.
